# Cell Therapy to Induce Allograft Tolerance: Time to Switch to Plan B?

**DOI:** 10.3389/fimmu.2015.00149

**Published:** 2015-04-07

**Authors:** Antoine Sicard, Alice Koenig, Emmanuel Morelon, Thierry Defrance, Olivier Thaunat

**Affiliations:** ^1^U1111, INSERM, Lyon, France; ^2^Hospices Civils de Lyon, Hôpital Edouard Herriot, Service de Transplantation, Néphrologie et Immunologie Clinique, Lyon, France; ^3^Université de Lyon, Lyon, France

**Keywords:** organ transplantation, cell therapy, tolerance, regulatory T cells, B cells, B10, Bregs

## Abstract

Organ transplantation is widely acknowledged as the best option for end stage failure of vital organs. Long-term graft survival is however limited by graft rejection, a destructive process resulting from the response of recipient’s immune system against donor-specific alloantigens. Prevention of rejection currently relies exclusively on immunosuppressive drugs that lack antigen specificity and therefore increase the risk for infections and cancers. Induction of donor-specific tolerance would provide indefinite graft survival without morbidity and therefore represents the grail of transplant immunologists. Progresses in the comprehension of immunoregulatory mechanisms over the last decades have paved the way for cell therapies to induce allograft tolerance. The first part of the present article reviews the promising results obtained in experimental models with adoptive transfer of *ex vivo*-expanded regulatory CD4+ T cells (CD4+ Tregs) and discuss which source and specificity should be preferred for transferred CD4+ Tregs. Interestingly, B cells have recently emerged as potent regulatory cells, able to establish a privileged crosstalk with CD4+ T cells. The second part of the present article reviews the evidences demonstrating the crucial role of regulatory B cells in transplantation tolerance. We propose the possibility to harness B cell regulatory functions to improve cell-based therapies aiming at inducing allograft tolerance.

## Introduction

Organ transplantation consists in the restoration of vital physiologic functions through the surgical substitution of a defective organ by a functioning graft retrieved from a donor. Because, the donor is from the same species but genetically different from the recipient, immune system of the latter will inevitably recognize donor-specific antigenic determinants (i.e., alloantigens) expressed by the graft, in particular, the highly polymorphic molecules from the human leukocyte antigen (HLA) complex. The alloimmune response that develops against donor-specific HLA molecules is responsible for tissue damages, which lead to the failure of the transplanted organ, a process named “rejection.”

Prevention of graft rejection currently, exclusively, relies upon immunosuppressive drugs, which have no antigen specificity. They act by preventing the activation of immune effectors or blocking cell proliferation (1), therefore, dampening immune responses. Not surprisingly, this global immune depression is responsible for major side effects for patients, in particular, increased risks for infectious diseases (2), and malignancies (3). Furthermore, because complete blockade of immune responses would kill the patients, therapeutic immunosuppression only partially block alloimmune responses. Although sufficient to slow down graft destruction, therapeutic immunosuppression does not fully prevent the development of chronic rejection, which remains the first cause of allograft loss (4).

In 1953, Medawar provided the first experimental evidence of a sustained alloantigen-specific unresponsiveness in the absence of chronic immunosuppression: a process that he named immune tolerance (5). Of all of the mechanisms involved in tolerance to allografts, which include deletion, anergy, ignorance, and clonal exhaustion, the role of active T-cell-mediated immunoregulation was long ago identified as being crucial (6). Not surprisingly, important efforts have therefore been developed in the clinic to harness regulatory CD4+ T cells (CD4+ Tregs) for inducing tolerance in both hematopoietic cell transplantation (7–9) and solid organ transplantation (10–13). A first strategy consists in promoting the expansion of CD4+ Tregs *in vivo* through immune interventions like co-stimulation blockade, alloantigen infusion, interleukin 2. These approaches are not addressed in the present review for the sake of brevity [for recent reviews, see Ref. (10, 12, 13)]. Alternatively, it has also been proposed to expand recipient’s CD4+ Tregs *ex vivo* for retransfer as cell therapy.

Beyond CD4+ Treg, other immune players play important roles in tolerance toward alloantigens. Some cells from the innate immune system (i.e., tolerogenic dendritic cells, regulatory macrophages, myeloid-derived suppressor cells) have been shown to display regulatory functions and their use has emerged as another promising strategy to induce tolerance (10, 14). Recently, the regulatory properties of B cells have also been recognized and identified as being essential in allograft tolerance (15, 16).

Focusing on approaches based on the adaptive immune system, we first provide an overview of data underlying the use of adoptive transfer of CD4+ Tregs to promote allograft tolerance. The possibility to harness regulatory properties of B cells is then discussed.

## Adoptive Transfer of CD4+ Tregs to Induce Allograft Tolerance

### T-cell-mediated immunoregulation

The concept of T-cell-mediated immunoregulation arose in the early 1970s, following the seminal description by Gershon and Kondo of thymic-derived lymphocytes able to suppress antigen-specific immune responses (6). Although regulatory activity has been reported for various T-cell subsets, including CD4+ IL-10-producing type 1 regulatory cells (17) and some CD8+ T cells (18), there is a wide consensus that T-cell mediated immunoregulation is enriched in the CD4+ Tregs subset.

CD4+ Tregs are classically identified by the co-expression of CD4 and interleukin-2 receptor α-chain (CD25) together with the transcription factor Forkhead box P3 (FOXP3) (19). While the latter is considered as the best phenotypic marker of CD4+ Tregs, it should be noted that FOXP3 is also expressed by CD8+ Treg and transiently in humans by non-regulatory activated T cells (20, 21). CD4+ Tregs have many other phenotypic characteristics that are non-specific and inconstant: expression of CD45RA, latency-associated peptide (LAP), glucocorticoid-induced TNFR-related protein (GITR), cytotoxic T-lymphocyte antigen-4 (CTLA-4), inducible costimulatory (ICOS) receptors for interleukin 1 (CD121a/b), and low expression of IL-7 receptor-α chain (CD127) (10, 22–25).

CD4+ Tregs are thought to exert their immunoregulatory functions through four complementary molecular mechanisms (26): (i) *secretion of inhibitory cytokines*, including interleukin-10 (IL-10), IL-35, and transforming growth factor-beta (TGF-β), which act on both conventional T cells and dendritic cells; (ii) *cytolysis* through CD95L, granzyme, and perforin-dependent killing mechanisms; (iii) *metabolic disruption*, which includes high-affinity CD25-dependent IL-2-deprivation-mediated apoptosis, consumption of extra-cellular ATP by CD39and/or CD73; and (iv) *mechanisms that modulate dendritic cell maturation and/or function* such as HLA-G, lymphocyte-activation gene 3 (LAG3; also known as CD223)–MHC-class-II mediated suppression of DC maturation, and cytotoxic T-lymphocyte antigen-4 (CTLA4)–CD80/CD86-mediated induction of indoleamine 2,3-dioxygenase (IDO), which catalyzes the tryptophan degradation forming the intermediate kinurenine with immunomodulatory properties.

### Naturally occurring vs. adaptive CD4+ Tregs

Part of CD4+ Treg efficiency comes from their ability to convert “conventional” T cells into cells with suppressive properties, a process referred to as infectious tolerance (27). One can therefore distinguish two categories of CD4+ Tregs, which differ in their origin, phenotype, and mode of action.

(i) Naturally occurring CD4+ Tregs or thymus-derived T reg (tTregs) that develop from T-cell precursors with some degree of self-reactivity during the normal process of T-cell maturation in the thymus, and survive in the periphery and are poised for immunoregulation.
(ii) Adaptive CD4+ Tregs that are generated extrathymically from CD4+ CD25-T-cells, either at peripheral sites *in vivo* [peripheral Treg (pTregs)], or induced in cell culture [*in vitro*-induced Treg (iTregs)] (28).

In contrast with T CD4+ Tregs, which are characterized by a complete demethylation of CpG motifs as well as histone modifications within regions of *FOXP3* locus, adaptive CD4+ Tregs display only incomplete demethylation that is lost, along with FOXP3 expression and suppressive activity upon restimulation in the absence of TGF-β (29). Higher stability of the regulatory phenotype of CD4+ tTregs suggests that they might be a better source for cell therapy than adaptive CD4+ Tregs, which may convert back into effectors after transfer into recipients due to their plasticity. However, this issue may not be as straightforward as it may seem because CD4+ tTregs are rare and difficult to separate from adaptive CD4+ Tregs alive, and also because CD4+ tTregs and adaptive CD4+ Tregs play complementary roles. In particular, recent observations made in mice, selectively lacking CD4+ pTregs, demonstrated their pivotal function in maternal tolerance toward paternally inherited fetal alloantigens (30), suggesting that adaptive CD4+ Tregs might be critical in the control of alloresponse in organ transplantation. Consequently, almost all cell therapy strategies to induce allograft tolerance rely on adoptive transfer of a mixture of CD4+ T Tregs and p/iTregs.

### Which specificity for transferred CD4+ Tregs?

CD4+ Tregs have been shown to maintain potent suppressive properties after *ex vivo* expansion (31, 32), suggesting that this strategy could be used to generate enough CD4+ Tregs as to alter the balance of T effector/Tregs in recipients after adoptive transfer and thus to induce tolerance to allograft. Accordingly, it was shown in a murine model of heart allotransplantation that purified polyclonal CD4+ Tregs, *ex vivo* expanded with anti-CD3/CD28 mAb-coated beads before reinfusion to recipient, efficiently prevented CD4+ T cell-mediated rejection and delayed CD4+/CD8+ T cell-mediated rejection (33). Using a similar strategy in a humanized mouse model, Kathryn Wood’s team reported that human *ex vivo* expanded polyclonal CD4+ Tregs were able to prevent the development of vascular chronic rejection lesions (34).

Based on these promising results, a clinical trial has been launched to evaluate the recipients’ CD4+ Tregs expanded polyclonaly (The ONE study: NCT02129881^1^).

An important limitation of this approach for clinical application is the fact that polyclonal CD4+ Tregs deliver pan-immunosuppressive effects, and therefore do not negate all the caveats associated with standard immunosuppression. Using donor-specific CD4+ Tregs could allow circumventing this problem.

**Figure 1:**
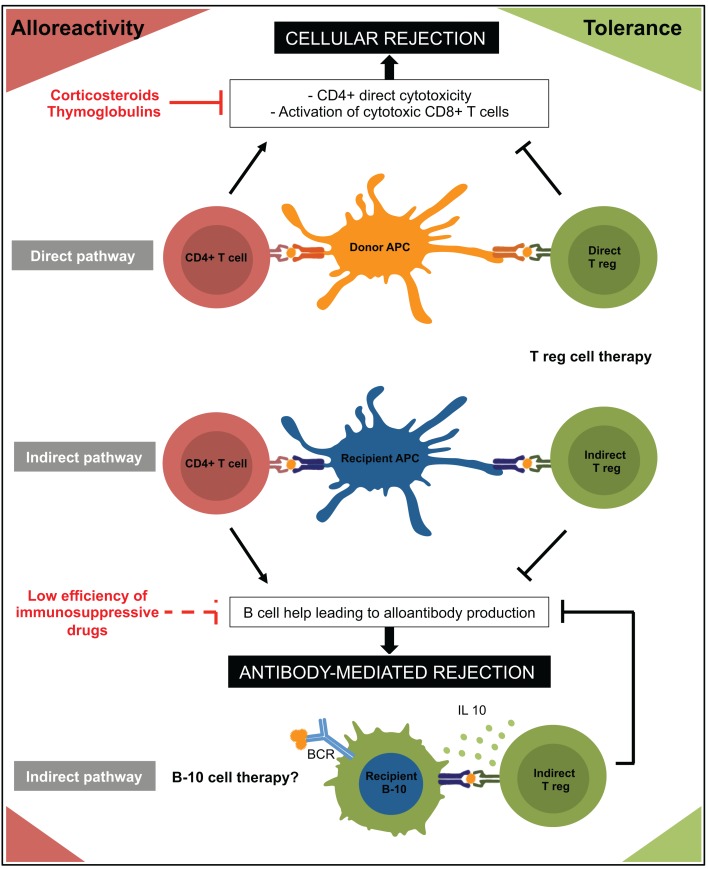
**Immune mechanisms involved in rejection and tolerance to allograft are represented**. Regulatory cells are in green and effector cells in red. APC, antigen presenting cell; BCR, B cell receptor.

Allorecognition is initiated by T cells recognizing either intact allo-MHC molecules on donor antigen-presenting cells (APCs) (direct pathway) or allopeptides bound to self-MHC molecules on recipient APCs (indirect pathway) (35). As for effectors, two type of donor-specific CD4+ Tregs can therefore participate to allograft tolerance: direct-CD4+ Tregs and indirect-CD4+ Tregs (Figure 1).

It is traditionally accepted that direct alloreactivity represents the driving force behind early acute graft rejection. Using donor-derived mature APCs to generate direct-CD4+ Tregs, Sagoo et al. reported that these “customized” CD4+ Tregs were more efficient than polyclonal CD4+ Tregs to prevent alloimmune-mediated injury of human skin grafts in a humanized mouse model (36). Several independent teams (37–41) reached similar conclusions using various protocols that all have in common the use of allogeneic APCs for *ex vivo* expansion of direct-CD4+ Tregs. These data paved the way for ongoing clinical trials aiming at evaluating alloantigen-specific direct-CD4+ Tregs in the setting of kidney transplantation (The ONE study: NCT02244801 and NCT02091232; see text footnote 1).

Interestingly, Joffre et al. demonstrated in a murine model that adoptive transfer of direct-CD4+ Tregs prevented acute rejection but failed to prevent the development of chronic rejection lesions on cardiac transplant (42). These findings are in line with the fact that, while direct alloresponse rapidly subsides as donor passenger leukocytes vanish, indirect alloresponse persists indefinitely and promotes chronic rejection (43). The importance of the indirect allorecognition pathway in allograft tolerance has been robustly demonstrated. B6II-4+ mice (MHC II-deficient mice expressing an MHC II transgene exclusively on thymic epithelium), which have functional CD4+ T cells but are unable to mount an indirect response, are resistant to the induction of cardiac allograft tolerance (44, 45). In humans, Haynes et al. showed that antidonor indirect pathway T regulatory response was much higher in operationally tolerant patients than in patients experiencing chronic rejection (46). All together, these data strongly suggest developing strategies of adoptive transfer of indirect-CD4+ Tregs to promote long-term allograft tolerance. In line with this hypothesis, several independent teams (using various methods to generate indirect-CD4+ Tregs) reported that these cells have a higher ability than direct-CD4+ Tregs to prolong allograft survival (42, 47, 48). Jiang et al. showed that this approach was transposable in the clinic (49). They recurrently stimulated CD4+ CD25+ T cells with irradiated autologous monocytes-derived dendritic cells pulsed with HLA-A2 peptide in presence of IL2 and IL7 to generate indirect-CD4+ Tregs. Interestingly, these cells not only suppressed antigen-driven responses of CD4+ CD25-T cells specific for the same peptide (indirect allorecognition) but also direct alloresponse of naive CD4+ CD25-T cells stimulated by semi-allogeneic APCs in the presence of the peptide (i.e., “linked suppression”).

### Indirect-CD4+ Tregs and *de novo* alloantibody generation

It is now widely recognized in organ transplantation that long-term graft loss is mainly caused by the recipient’s humoral response against donor HLA molecules (50). The binding of donor-specific antibodies (DSA) to mismatched HLA molecules leads to chronic microvascular inflammation and progressive tissue destruction through complement activation and antibody-dependent cell cytotoxicity (51–56).

Donor-specific antibodies are produced upon activation of recipient’s alloantigen-specific B cells (57). The binding of cognate alloantigen to B cell receptor (BCR) provides the first activation signal to B cell, and leads to the internalization, processing, and presentation of the alloantigen on surface MHC class II molecules. Cognate CD4+ T lymphocytes, which recognize the alloantigen indirectly presented by B cells, provide the second activation signal through provision of CD40L. Activated B cells then either differentiate into plasmablasts secreting low affinity IgM or undergo the germinal center reaction driven by cognate interactions with a particular subset of CD4+ T cells: the follicular helper T cells (58). After affinity maturation and isotype switching, B cells differentiate either into memory B cells or into long-lived plasma cells, which secrete high affinity switched DSA and localize into the spleen and the bone marrow (59). CD4+ T cells specific for indirectly presented alloantigens are therefore necessary for the generation of DSA. This was first showed experimentally by Steele et al. (60) and then robustly confirmed by Pettigrew’s team (61–64). Accordingly, a patient from the ITN507ST trial, who exhibited a weak indirect pathway response to HLA-A1 and HLA-B57 but a strong response to HLA-A2, also developed an anti-HLA-A2 IgG response but no detectable Ab response to HLA-A1 and HLA-B57 (46).

Based on these data, it is reasonable to speculate that the long-term beneficial effect reported for adoptive transfer of indirect-CD4+ Tregs is due to their ability to suppress DSA generation (Figure 1). In accordance with this hypothesis, Callaghan et al. showed in a rat transplant model that the transfer of indirect CD4+ Tregs prevented alloantibody production (65).

An important limitation to the transfer in the clinic of protocols of cell therapy with *ex vivo*-expanded indirect-CD4+ Tregs is the source of recipient’s APCs. Indeed, dendritic cells are rare in the circulation and monocytes require several days of maturation in culture to differentiate into mature APCs. In contrast with their deleterious role in alloantibody production, B cells are also endowed with regulatory functions (15, 16). As mentioned above, they are also potent APCs, which establish a privileged crosstalk with CD4+ T cells. Finally, they are in greater number in the circulation than professional APCs and are also easy to expand and to keep *in vitro* upon CD40 stimulation. For all these reasons, B cells might represent promising generators of indirect-CD4+ Tregs.

## Harnessing B Cell Regulatory Functions to Induce Allograft Tolerance

### Regulatory function of B cells

One of the first evidence that B cells could regulate immune responses came from an experimental study published in 1982, which reported that antigen-activated B cells could suppress immune responses *in vivo* (66). Later on, many studies conducted in various experimental models of immune diseases confirmed the strong contribution of B cells to immune regulation (67–69). For instance, Wolf et al. reported that mice lacking B cells developed a more severe form of experimental autoimmune encephalomyelitis (EAE) (70). Similar observations were made in murine experimental models of collagen induced arthritis (71), ulcerative colitis (72), and allergy (73, 74). Interestingly, though the pathogenic T-cell response involves the same T helper 1 (Th1) cells and Th17 proinflammatory T-cell populations in EAE and collagen-induced arthritis, the colitis and allergy models differ in that the inflammation appears to be driven by Th2 cells. Thus, the B-cell compartment has the capacity to control organ-specific inflammation that may be driven by Th1, Th2, or Th17 effectors.

Beyond murine experimental models, accumulating evidence from patients with multiple sclerosis (75), lupus (76), and rheumatoid arthritis (77) suggests that B cells play a crucial regulatory role also in humans.

### B cell regulation in clinical transplantation

The demonstration that B cells play a major regulatory role in clinical transplantation came from studies performed in the few spontaneously operationally tolerant patients, i.e., kidney recipients who have kept a stable graft function for years after immunosuppression withdrawal (15). Brouard et al. were the first to report that operationally tolerant kidney recipients could be identified by a blood transcriptional signature enriched for B cell related genes (78). This seminal finding has been further validated by two independent studies from Immune Tolerance Network (79) and the Indices of Tolerance European Union consortium (80), which demonstrated that tolerant, but not stable patients under immunosuppression exhibited enriched B cells and B cell transcripts in their blood. Cross-validation studies in these two consortia confirmed a strong association between B cell-related genes/markers and the tolerant state. Interestingly, while neither group performed transplant kidney biopsies, RNA from cells in the urine in the US cohort of tolerant patients contained higher quantities of CD20 transcripts, suggesting that B cells in the graft may be relevant (81).

### Mechanisms of B cell-mediated regulation

Using antibodies against CD45, a tyrosin–phosphatase involved in lymphocyte activation, Deng et al. induced tolerance to allogeneic heart in mice (82). This therapy was ineffective in recipients lacking B cells (μMT). Reconstitution of μMT recipients with B cells incapable of antibody secretion was sufficient to restore tolerance but this effect was lost if the reconstitution was made with B cells from CD40 or B7-1/B7-2 knockout mice. In another study, Ding et al. showed that tolerance to allogeneic islet could be induced with anti-TIM1 antibodies only if B cells were present in the recipient (83). In this model, the transfer of B cells from B6-immunized BALB/c mice abrogated rejection of B6 but not C3H islets by BALB/c recipients. Combining anti-CD45+ anti-TIM1, Lee et al. (84) observed a synergistic effect for inducing tolerance to allogeneic islets. This effect depended on the presence of recipient B cells and was antigen-specific. Tolerance could not be induced if Tregs were depleted in recipient. Collectively, these *in vivo* murine experimental studies prove that B cells have the capacity to transfer donor-specific tolerance. They also demonstrate the importance of the crosstalk between host B and T cells in B cell-mediated transplantation tolerance.

The identification of the molecular mechanisms by which B cells exert their regulatory functions on T cells has been the subject of considerable interest. Although B cells have been shown to induce T effector apoptosis by up regulation of FAS-Ligand or production of granzyme B, the main regulatory mechanism appears to be the secretion of immune-regulatory cytokine: IL35 (85), TGF-β, and primarily IL10 (69, 74) (Figure 1). Fillatreau et al. indeed showed that chimeric mice in which IL10 deficiency was restricted to B cells did not recover from EAE and had a persistent Th1 inflammatory response (86). The IL10-mediated suppressive function of B cells was also demonstrated in colitis, arthritis, and allergy models (71, 74, 87). IL10 has pleiotropic effects on immune responses. While restraining inflammation, inhibiting dendritic cells or CD8+ T cells functions and suppressing Th1 and Th17 cells responses, IL10 also promotes Tregs (and Tr1) differentiation (88).

### IL10-producing B cells: “B10” cells

The molecular signals driving IL10 production in B cells have been partly identified. TLR signaling is determinant (89). Deficiencies in TLR2 and TLR4 or in MyD88 (the adapter used by most TLRs to activate the transcription factor NF-κB) restricted to B cells were associated with a decrease production of IL10 and the development of a severe form of EAE (90).

B cell receptor signaling also plays a critical role in IL10 production by B cells. Deficiency in B-cell linker protein (BLNK), an upstream adapter in the BCR pathway, indeed profoundly decreased IL10 production (91). In line with this finding, impaired calcium signaling in STIM1 and STIM2 deficient mice also weakened IL10 production by B cells (92).

CD40 pathway is also involved. Mice with a CD40 deficiency, which is restricted to B cells, developed a more severe EAE form with important decrease of IL-10 production (86).

Because these three pathways are intermingled during B cell activation, it is difficult to determine their specific contribution to *in vivo* B cell IL10 production. Tedder’s team suggested that the generation of IL-10-secreting B cells required IL-21 and CD40-dependent cognate interactions with T cells (93). Fillatreau’s group proposed a two-step model in which B cells-mediated regulation is initiated by TLR ligation and then strengthened by BCR and CD40 signaling (68, 89).

The involvement of the B-cell receptor, CD40, and TLRs in the regulatory function of B cells raises a conceptual difficulty. Indeed, these signals are the very same as the ones involved in the activation of B cells in most immune responses. One hypothesis would therefore be the existence of a peculiar “Breg” subset, endowed with the unique function to regulate immune processes. In the mouse, a rare population of splenic B cells characterized by a unique phenotype (CD5+ CD1d high) has been reported to play a critical role in the regulation of murine EAE (94). Other groups have since ascribed this role to a wide range of B cell subsets, including peritoneal B-1 cells, marginal zone B cells, transitional type 2 B cells (76), TIM-1+ (83). More recently, it has been suggested that plasmablasts could also exert regulatory function through provision of IL10 (95).

The diversity of B-cell subsets involved in suppression raises an alternative hypothesis: instead of being a unique property of a single B-cell subset, immune-suppressive activity could be exerted by different subsets of B cells depending on the integration of available signals in the microenvironment.

### *Ex vivo*-generation of autologous B10 for cell therapy

Two important features have to be met by B cells for cell therapy aiming at inducing immune tolerance. First, B cells must display regulatory functions. This is made possible by providing them with activation signals inducing the synthesis of IL10 (described in detail above). The feasibility of this approach was demonstrated by Lampropoulou et al., who used TLR ligands to generate B10 *in vitro* able to suppress T cell activation (90). Of note, in contrast with CD4+ Tregs, the stability of regulatory functions of which might be compromised following transfer by immunosuppressive drugs or by graft inflammatory environment (89, 96), danger signals (signaling through TLR) present within the graft could participate to maintain the regulatory functions of B cells.

A second important feature for *in vitro*-generated B10 is their ability to present the relevant antigen in MHC class II molecules in order to efficiently interact with cognate T cells (89, 97). This point is far more challenging. Indeed, in contrast with dendritic cells that present any antigens following phagocytosis, cognate interaction with the BCR is required for antigen internalization, processing, and presentation by B cells (98). For this reason, it is difficult to pulse polyclonal populations of B cells with a given protein antigen. To overcome this limitation, Scott’s group has developed an original approach consisting in transducing polyclonal B cells with a retroviral vector encoding for the antigen (99). Building on this technology, his group has made pioneer contributions highlighting the potential of B lymphocytes for cell therapy to induce antigen-specific tolerance (100, 101). In Scott’s system, antigen is retrovirally transduced into LPS-activated B cells. Upon adoptive transfer, genetically modified B cells were able to inhibit autoimmune diseases in many mice models including uveitis (102, 103), multiple sclerosis (104, 105), type 1 diabetes (106), and rheumatoid arthritis (107). The expression of MHC II and co-stimulatory molecules (B7.1 and B7.2) by transduced B cells was necessary, highlighting again the importance of B cell antigen presentation to T cells for tolerance (108–110). Genetically engineered B cells were indeed shown to act by promoting the expansion of FOXP3+ CD4+ Tregs *in vivo* (111). It is not clear in Scott’s system why the transduced antigen was expressed in the MHC II of genetically engineered B cells. This could be due to the fact that antigen was engineered in frame into BCR, which traffic through endosomal compartment.

Fillatreau’s group recently showed that it was possible to transduce *in vitro* resting B cells with lentiviral vectors encoding for the oligodendrocyte glycoprotein (MOG) and the IL10 genes. These reprogramed quiescent B cells efficiently protected against EAE upon adoptive transfer in mice (112).

Thus, B cell gene therapy for tolerance induction is associated with excellent results in murine models of autoimmune diseases. However, further work is needed to assess whether it is transposable to allograft tolerance and to human B cells that exhibit phenotypic differences with their murine counterpart (69). The behavior of the genetically modified B10 cells *in vivo* has to be better characterized: are their lifespan, regulatory functions, or proliferative capacity modified? Do they maintain their immunosuppressive and proliferative properties under the immunosuppressive drugs classically used in organ transplantation? The question of their phenotypic stability overtime is primordial given the risk to promote allograft rejection instead of tolerance. It shall also be kept in mind that the use of retroviral and lentiviral vectors raises concerns about safety in humans. Indeed, the possibility of oncogenic consequences caused by random insertions in the human genome cannot be totally excluded in such approaches (99). Practical concerns would mainly lie in the requirement for sophisticated skills and for authorizations to use genetically modified cells and in the size of the inserts coding for antigenic sequences that are limited to approximately 10 Kb (112).

## Conclusion and Perspectives

CD4+ Tregs therapy induces tolerance to allogeneic transplant in various murine models. These experimental data have paved the way for ongoing clinical studies, which will determine the effect of adoptive transfer of *ex vivo*-expanded polyclonal or donor-specific direct-CD4+ Tregs in kidney recipients.

Experimental studies have nevertheless demonstrated that direct-CD4+ Tregs are insufficient to promote long-term allograft survival, which requires CD4+ Tregs with indirect specificity for donor antigens. The transfer in the clinic of protocols for *ex vivo* expansion of indirect-CD4+ Tregs faces, however, a difficult challenge: the source of recipient’s APCs.

Recently, Landwehr-Kenzel et al. (113) described a GMP-compliant protocol for expansion of direct-CD4+ Tregs using an allogeneic B cell bank. This strategy could serve as a base for the use of recipient’s B cells to generate indirect-CD4+ Tregs. B cells are indeed found in number in the circulation, they don’t need maturation, and are easy to expand and to keep *in vitro* upon CD40 stimulation. More importantly, they are potent APCs for which the molecular signals controlling IL10 production (and therefore regulatory functions) are known. Such donor-specific B10 could be used to induce tolerance directly (B10 cell therapy) or indirectly (for *ex vivo* expansion of indirect-CD4+ Tregs). An important hurdle remains however: in contrast with dendritic cells, B cells cannot perform phagocytosis. Only cognate antigen able to bind to BCR is spontaneously internalized and presented in MHC II. The use of B cells as tolerogenic APCs therefore requires designing safe strategies to pulse them with donor antigens.

## Conflict of Interest Statement

The authors declare that the research was conducted in the absence of any commercial or financial relationships that could be construed as a potential conflict of interest.

## Footnotes

^1^https://clinicaltrials.gov
